# Filling Exciton Trap-States in Two-Dimensional Tungsten Disulfide (WS_2_) and Diselenide (WSe_2_) Monolayers

**DOI:** 10.3390/nano11030770

**Published:** 2021-03-18

**Authors:** Zeynep Ezgi Eroglu, Dillon Contreras, Pouya Bahrami, Nurul Azam, Masoud Mahjouri-Samani, Abdelaziz Boulesbaa

**Affiliations:** 1 Department of Chemistry & Biochemistry, California State University, Northridge, 18111 Nordhoff Street, Northridge, CA 91330, USA; zeynep.eroglu.568@my.csun.edu (Z.E.E.); dillon.contreras.3@my.csun.edu (D.C.); pouya.bahrami.60@my.csun.edu (P.B.); 2 Department of Electrical and Computer Engineering, Auburn University, Auburn, AL 36849, USA; mna0018@auburn.edu (N.A.); mzm0185@auburn.edu (M.M.-S.)

**Keywords:** 2D materials, TMDs, excitons, defects, ultrafast dynamics

## Abstract

Two-dimensional transition metal dichalcogenides (2D-TMDs) hold a great potential to platform future flexible optoelectronics. The beating hearts of these materials are their excitons known as X_A_ and X_B_, which arise from transitions between spin-orbit split (SOS) levels in the conduction and valence bands at the K-point. The functionality of 2D-TMD-based devices is determined by the dynamics of these excitons. One of the most consequential channels of exciton decay on the device functionality is the defect-assisted recombination (DAR). Here, we employ steady-state absorption and emission spectroscopies, and pump density-dependent femtosecond transient absorption spectroscopy to report on the effect of DAR on the lifetime of excitons in monolayers of tungsten disulfide (2D-WS_2_) and diselenide (2D-WSe_2_). These pump-probe measurements suggested that while exciton decay dynamics in both monolayers are driven by DAR, in 2D-WS_2_, defect states near the X_B_ exciton fill up before those near the X_A_ exciton. However, in the 2D-WSe_2_ monolayer, the defect states fill up similarly. Understanding the contribution of DAR on the lifetime of excitons and the partition of this decay channel between X_A_ and X_B_ excitons may open new horizons for the incorporation of 2D-TMD materials in future optoelectronics.

## 1. Introduction

During the last decade, an extensive research effort has been devoted to the investigation of the physical, electrical, and optical properties of two-dimensional transition metal dichalcogenide materials (2D-TMDs) [1,2,3,4,5,6]. This is not surprising when taking into account the potential of these materials to revolutionize various applications, ranging from photovoltaics [7,8,9], to sensing [10], to information technology [11]. A common pillar between these applications is the necessity of using materials that strongly interact with light and have excitons with special properties. Monolayers of 2D-TMDs absorb light up to 10% within the solar spectrum [9] and possess excitons that diffuse for long ranges [12,13,14], reaching ~150 nm in the case of tungsten diselenide (WSe_2_) [14]. These properties originate from the extraordinarily strong Coulomb interaction resulting from the high geometrical confinement, weak dielectric screenings, and the indirect-to-direct gap transition when the thickness is reduced to a single monolayer [3,15,16]. Some of the consequences of the excitons being in 2D-TMDs are that they are tightly bound, which diminishes the conversion efficiency of devices where exciton dissociation is required [17,18]. Another consequence is the susceptibility of exciton to traps due to defects induced to monolayers during their chemical synthesis or physical exfoliation. To understand the effect of defects on excitons, one excellent strategy consists of investigating their dynamics via photoluminescence (PL) and transient absorption spectroscopy techniques.

The two excitons of particular interest in 2D-TMDs are commonly referred to by X_A_ and X_B_, which arise from transitions between spin-orbit split (SOS) levels at the conduction band (CB) and valence band (VB) at the K-point [19]. Several previous studies of exciton dynamics in 2D-TMD monolayers have indicated that the lowest exciton decays through fast and slow channels [20,21,22,23]. For instance, temperature and pump fluence-dependent studies of exciton dynamics suggested that thermalisation and relaxation take place within the first ~2 ps, whereas defect-assisted recombination (DAR) is a slower decay-channel [21,22]. We note that the excitation of 2D-TMD monolayers leads not only to the photogeneration of excitons, but to free charge carriers as well, notably when the excitation energy is well above the band-gap [24,25]. These free charge carriers are more vulnerable for traps than excitons. Time-resolved (TR) PL in tungsten disulfide (WS_2_) indicated that the decay of excitons strongly depends on excitation density due to exciton-exciton annihilation, which is faster by two orders of magnitude in monolayers compared to bi- and tri-layers [6]. In fact, studies of excitation density-dependent TRPL conducted on WSe_2_ monolayers suggested that during the first few picoseconds following excitation, the PL decay is dominated by multiexciton interactions [26]. At later times after the photoexcitation, studies of temperature and pump fluence-dependent dynamics of charge carriers in molybdenum disulfide (MoS_2_) monolayers suggested that most electron-hole pairs recombine through the DAR pathway [21].

Here, we employ steady-state absorption and PL spectroscopies to identify the excitonic transitions X_A_ and X_B_, and femtosecond pump-probe spectroscopy to study the dependence of ultrafast dynamics of exciton decay on the excitation density in 2D-WS_2_ and 2D-WSe_2_ monolayers. These dynamics are measured through monitoring the exciton depletion recovery following excitation at ~400 nm (~3.1 eV). For both studied materials, pump-probe results indicated that the excited population decays through a fast component during the first few picoseconds after excitation, and the remaining population decays through a slower pathway that takes place from tens to hundreds of picoseconds. According to previous reports, this slow decay pathway is attributed to DAR, which is found to dominate the decay of both excitons in the studied monolayers. Furthermore, these pump-probe measurements suggested that in 2D-WS_2_, defect states near the X_B_ exciton fill-up before those near the X_A_ exciton; however, in the 2D-WSe_2_ monolayer, the defect states fill up similarly. Understanding the contribution of DAR to the lifetime of excitons, and the partition of this decay channel between X_A_ and X_B_ excitons may open new perspectives for the incorporation of 2D-TMD materials in future optoelectronics.

## 2. Materials and Methods

### 2.1. Subject Material

The laser-assisted synthesis technique (LAST) followed to prepare 2D-WS_2_ and 2D-WSe_2_ monolayer crystals has been previously described [27]. Briefly, a continuous-wave CO_2_ laser emitting at 10.6 µm wavelength is used to heat and evaporate stoichiometric powders placed inside a graphite boat (1.2 × 0.7 × 0.7 cm^3^) during 90 s with 35 W power. Fused silica substrates are placed upside down at a distance of 6 mm right above the graphite boat to capture the vapor. To ensure a favorable growth environment for 2D-WS_2_ and 2D-WSe_2_ monolayers, the substrate and the graphite boat are placed inside a 1-inch tube furnace. To evacuate air and chemical residuals, the tube furnace is pumped down to a few millitorrs before starting the synthesis process. Subsequently, Argon gas is flown through the tube furnace maintaining the background pressure around 150 Torr during the growth process at 750 °C temperature.

### 2.2. Femtosecond Transient Absorption Spectroscopy

Femtosecond transient absorption spectroscopy measurements are carried out using the experimental setup described in our previous report [24]. Briefly, it is based on a Ti-Sa femtosecond amplifier (Astrella by Coherent Inc., Santa Clara, CA, USA). This laser source provides ~35 fs short pulses centered at 800 nm with an average power of 6 W at a repetition rate of 5 kHz. About 1 W of the amplifier’s output is used to generate pump pulses at 400 nm (3.1 eV) in a 0.5 mm thin beta barium borate (BBO) crystal by frequency-doubling the 800 nm fundamental laser. To maintain the pulse’s ultrashort duration, the frequency doubling is done without focusing and collimating lenses, and the filtration of the residual 800 nm is achieved using a filter that reflects the 400 nm and transmits the 800 nm. The probe pulse is a spectrally broad (460–920 nm) white light continuum (WL) generated by focusing a small portion of the Astrella’s output onto a 2 mm thick sapphire window. A reflective parabolic mirror is used for the collimation of the WL, and a reflective filter (transmits 800 nm and reflects other wavelengths) is used for the filtration of the 800 nm fundamental laser to avoid optical chirp in the spectrally broad WL. The pump and probe beams are brought collinearly to the input of a home-built inverted-upright hybrid microscope using a thin (0.5 mm) dichroic filter (which transmits the WL probe and reflects the 400 nm pump). Using a 40× reflective objective microscope, the pump and probe beams are focused on the sample down to ~<5 μm spot sizes. After the sample, the transmitted probe is collimated using a 35 mm focal-length calcium fluoride (CaF_2_) lens and focused onto a 100 μm slit entrance of a spectrograph (iHR320 by Horiba Scientific, Piscataway, NJ, USA), which is coupled with a CCD (Andor Newton by Oxford Instruments, Abingdon, UK) that is equipped with an electron multiplier (EM).

In order to control the time-delay between the pump and the probe pulses, the pump beam passes through a motorized stage (MIMS600CC by Newport Corporation, Irvine, CA, USA). The pump power is controlled using a rotating variable optical density filter. To cancel out long-term laser fluctuations, the pump beam passes through an optical chopper set to a frequency of 100 Hz, and the absorbance change (ΔA) at every time-delay is calculated between every 50 successive laser shots. In every experiment, three scans over the covered time-delay range (1 ns) are averaged.

### 2.3. Steady-State Absorption and Emission Spectroscopy

The home-built upright-inverted microscope is equipped with a tungsten halogen light source (HL-2000-LL by Ocean Insight, Orlando, FL, USA) that provides a stable and spectrally broad white-light (360–900 nm). This light is used for measuring steady-state absorption spectra from the same 2D monolayer crystals used in pump-probe experiments. For the steady-state emission measurements, the microscope is equipped with a continuous-wave (CW) laser source emitting at ~400 nm, which is used for exciting the same 2D monolayer crystals used in pump-probe experiments. For both steady-state emission and absorption experiments, the optical paths after the sample and the detection system are the same as those used in transient absorption measurements.

## 3. Results and Discussion

**Figure 1:**
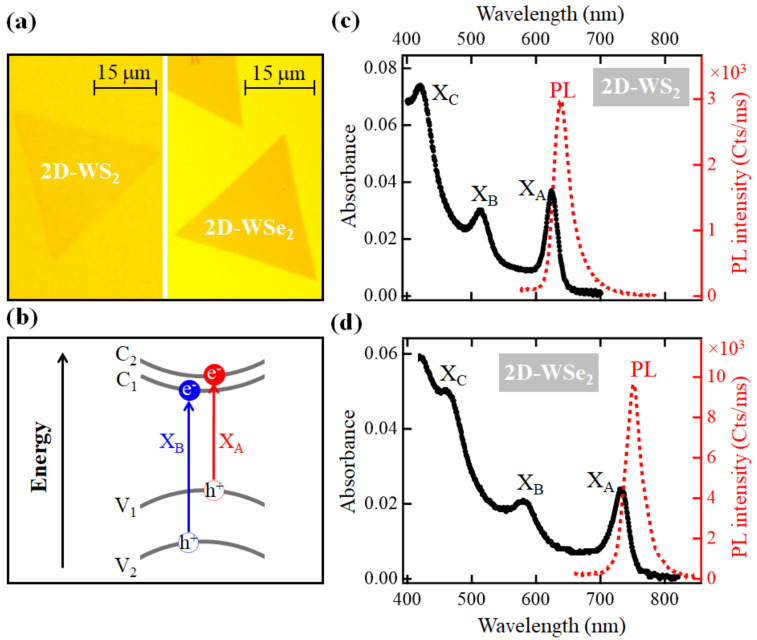
(**a**) Optical images of the studied monolayer single crystals of 2D-WS_2_ and 2D-WSe_2_; (**b**) depiction of the excitonic transitions X_A_ (V_1_ → C_2_) and X_B_ (V_2_ → C_1_) between the spin-orbit split levels at the VB and CB in 2D-TMDs; (**c**) steady-state absorption spectrum measured at the 2D-WS_2_ monolayer crystal shown in (**a**). The dotted line is the PL spectrum measured following excitation at ~400 nm; (**d**) steady-state absorption spectrum measured at the 2D-WSe_2_ monolayer crystal shown in (**a**). The dotted-plot is the PL spectrum measured following excitation at ~400 nm.

Optical images of the studied monolayer crystals of 2D-WS_2_ and 2D-WSe_2_ are shown in Figure 1a. These crystals are about 30 μm wide, and they are sufficiently large for spectroscopy/microscopy studies considering that laser spot sizes focused on the sample are smaller than 5 μm. The SOS levels in the VB and CB and the corresponding allowed excitonic transitions X_A_ and X_B_ [19] are depicted in Figure 1b. The SOS split levels V_1_ and V_2_ in the VB are separated by ~400 meV and ~500 meV for 2D-WS_2_ and 2D-WSe_2_, respectively [19,28]. In the CB, the splitting is about an order of magnitude smaller where the corresponding levels C_1_ and C_2_ are separated by about 15 meV and 40 meV for 2D-WS_2_ and 2D-WSe_2_, respectively [29]. Among the four possible transitions at each K^+^ (K^−^) space between these SOS levels, only two transitions are allowed at each K^+^ (K^−^) space. Electrons brought from the V_1_ level in the VB to the C_2_ level in the CB form the lowest energy exciton X_A_, and electrons transitioning from the V_2_ level in the VB to the C_1_ level in the CB make the X_B_ exciton.

Steady-state absorption spectra measured from the studied 2D-WS_2_ and 2D-WSe_2_ monolayer crystals are shown in Figure 1c,d, respectively. In the case of 2D-WS_2_, the absorption peaks of X_A_ and X_B_ excitons are centered at ~620 nm (~2 eV) and ~515 nm (~2.4 eV), respectively. For the 2D-WSe_2_ monolayer, the peaks of these excitons are centered at ~730 nm (1.69 eV) for X_A_ and at ~580 nm (~2.13 eV) for X_B_. The additional high energy peak that appears at ~420 nm (~2.95 eV) and at ~460 nm (~2.7 eV) for 2D-WS_2_ and 2D-WSe_2_ monolayers, respectively, are assigned to the X_C_ exciton, which originates from transitions near the Λ point in the space due to the nesting effect [25,30,31].

To verify the monolayer character of the studied crystals, steady-state PL spectra are collected following a CW laser excitation at ~400 nm. As shown in Figure 1c,d, the PL peaks are intense and sharp, indicating the direct K-K transition, which is characteristic of monolayers [32]. The PL peaks are centered at ~635 nm (~1.95 eV) and ~750 nm (~1.65 eV) for 2D-WS_2_ and 2D-WSe_2_, respectively.

The strategy that is followed to investigate the filling dynamics of trap-states consists of exciting the monolayer crystals with photon energies well above their band-gaps with different pump densities, then probing the decay dynamics of the lowest excitons X_A_ and X_B_. The excitation photon-energy used in pump-probe experiments is ~3.1 eV (~400 nm), which is well above the band-gaps of 2D-WS_2_ (~2 eV) and 2D-WSe_2_ (~1.65 eV) monolayers. This excitation allows the promotion of electrons from levels deeper than V_1_ and V_2_ in the VB to energy levels higher than C_1_ and C_2_ in the CB. Consequently, during their intraband relaxations, these excited electrons and holes are susceptible to trapping, not only by defect sites located within the band gap, but also by trap states located within the CB and VB of these monolayers.

**Figure 2:**
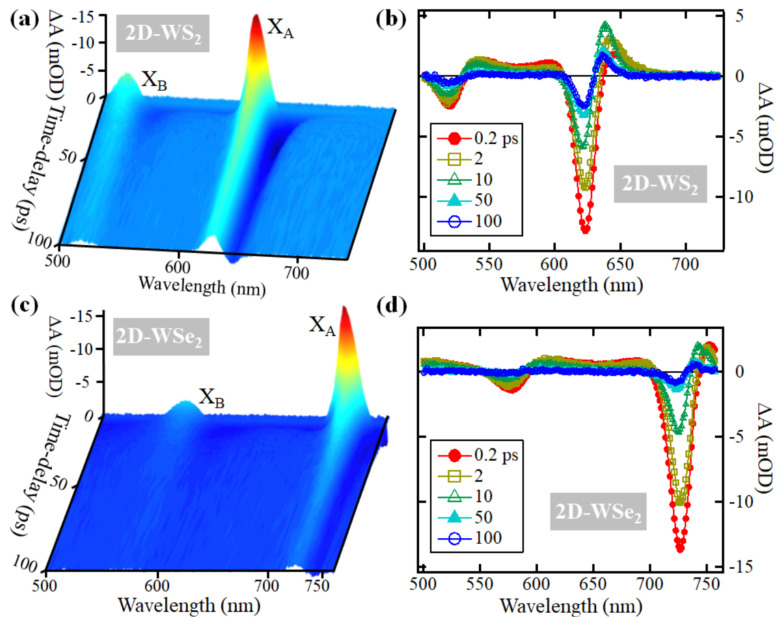
(**a**) Transient absorption results following excitation at ~400 nm (~3.1 eV) with a fluence of ~4 μJ·cm^−2^ of a single crystal of 2D-WS_2_ monolayer. (**b**) Spectral cuts at different time-delays from (**a**). (**c**) Transient absorption results following excitation at ~400 nm (~3.1 eV) with a fluence of ~2.8 μJ·cm^−2^ of a 2D-WSe_2_ single crystal monolayer. (**d**) Spectral cuts at different time-delays from (**c**).

To display the general dynamic spectral features that manifest in pump-probe experiments conducted on 2D-WS_2_ and 2D-WSe_2_ monolayers, we show in Figure 2 the absorbance changes (ΔA) recorded up to 1 ns following an excitation at ~400 nm (~3.1 eV). In the case of the 2D-WS_2_ monolayer, the transient absorption features that are shown in Figure 2a,b contain two negative bands around 510 nm and 620 nm that are superimposed on a spectrally broad band with weaker positive amplitude. Similarly, in the case of the 2D-WSe_2_ monolayer, Figure 2c,d show two negative peaks around 580 nm and 735 nm that are superimposed on a spectrally broad band with weaker positive amplitude. Based on the steady-state absorption spectra shown in Figure 1c,d, the negative peaks observed in the transient absorption spectra correspond to the ground state depletions of excitons X_A_ and X_B_. Because these depletion signals span a range of ~100 meV, the contribution of dark exciton transitions V_1_ → C_1_ and V_2_ → C_2_, which are separated from the bright transitions by only ~15–30 meV, is included in the depletion signals. We note that upon excitation by the pump pulse, the ground-state levels V_1_ and V_2_ are depleted through the X_A_ and X_B_ transitions, respectively. When the probe pulse arrives, the sample does not absorb at the wavelengths of X_A_ and X_B_ because the transitions have already happened due to the absorption of the pump. Because at later time-delays some electrons have recombined with holes, the observed depletion signal recovers back to zero (V_1_ and V_2_ levels are occupied again), and this is called depletion recovery.

The positive broad bands are absorptions induced by the pump, and they originate from several effects. For example, in molecular systems, these induced absorptions are due to the absorption of probe photons by the excited-state of the molecule (excited by the pump). In semiconductors, excitons and electrons excited by the pump may absorb probe photons at different energies to transition to higher energy excitons and levels in the CB, respectively [33]. Additionally, these induced absorptions may arise from many-body effects that manifest as peak broadening mechanisms such as multiple exciton generation [34]. Another possible origin of the positive induced absorption consists of the formation of charged excitons (trions) due to the trapping of electrons and/or holes at states that belong either to the substrate or intrinsic defects in the monolayer [19,35]. Because many-body effects manifest mostly at early time-delays [6,34,36,37], and the scope of this work concerns the defect assisted recombination, which is a slow process, we conducted a multi-peak fitting of the transient spectra taken at 10 ps delay shown in Figure 2b,d to characterize the different spectral features present in the positive broad induced absorption band. This analysis is presented in Figure S1, and the fitting parameters are listed in Tables S1 and S2 for 2D-WS_2_ and 2D-WSe_2_, respectively, in the Supplementary Materials.

**Figure 3:**
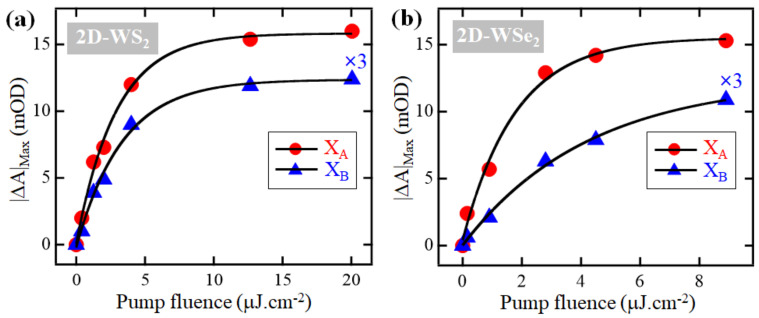
Dependence of the depletion signal maximal amplitudes of excitons X_A_ and X_B_ (magnified by 3) on the excitation fluence (symbols): (**a**) For 2D-WS_2_; (**b**) For 2D-WSe_2_. Solid lines are fits with a single exponential growth function to guide the eye.

Because at early time-delays following the excitation of the monolayers the maximum amplitudes of exciton depletion signals are proportional to the number of photogenerated excitons and free charge carriers, we plot in Figure 3 the dependence of the depletion maxima for excitons X_A_ and X_B_ on the fluence of the excitation of 2D-WS_2_ and 2D-WSe_2_ monolayers. In either of the monolayers and for both excitons, the relationship appears to be linear until the pump density is about 4 and 2.8 μJ·cm^−2^ for 2D-WS_2_ and 2D-WSe_2_, respectively. Beyond these values, the signal amplitudes—and thus the number of photogenerated excitons and free charge carriers—reach saturation; this implies that most, if not all, available exciton and free charge carrier states are filled. These excited electrons and holes are susceptible to trapping by defect-states located within the band-gap, in the CB, or in the VB.

**Figure 4:**
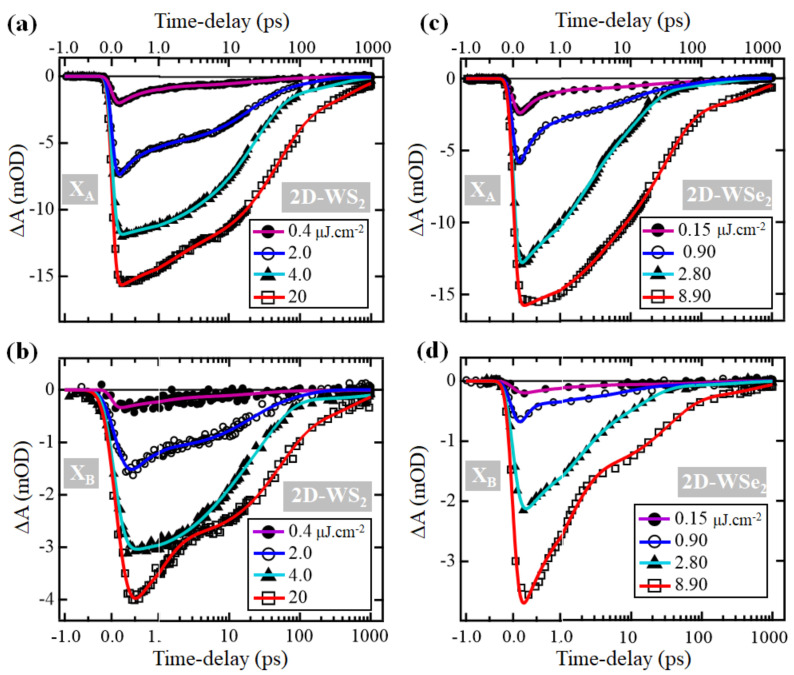
Ultrafast dynamics of exciton depletion signals following excitation at ~3.1 eV (400 nm) with different pump densities. (**a**) Exciton X_A_ depletion averaged around 620 nm in 2D-WS_2_. (**b**) Exciton X_B_ depletion averaged around 510 nm in 2D-WS_2_. (**c**) Exciton X_A_ depletion averaged around 735 nm in 2D-WSe_2_. (**d**) Exciton X_B_ depletion averaged around 595 nm in 2D-WSe_2_. Solid lines are fits to a triexponential decay function convoluted with a Gaussian instrument response function of 45 fs width. The time-delay axes are shown in linear scale up to 1 ps, and logarithmic scale thereafter.

To obtain information about the exciton trapping dynamics, we examine the dependence of the depletion recovery dynamics of excitons X_A_ and X_B_ on the excitation density as shown in Figure 4. These decay traces are fit to a tri-exponential decay function convoluted with a 45 fs Gaussian instrument response function (IRF), and the returned parameters of the converged fits are listed in Table 1 and Table 2 for 2D-WS_2_ and 2D-WSe_2_ monolayers, respectively. In the case of the 2D-WS_2_ monolayer, the amplitude-weighted average lifetime $T_{ave}$ for the X_A_ exciton increases linearly with increasing pump density; however, in the case of 2D-WSe_2_, an increase of $T_{ave}$ with increasing excitation density is observed only after the second pump density. In the case of the X_B_ exciton, for both studied monolayers, a linear dependence of $T_{ave}$ on the pump fluence is not observed.

The dynamics of X_B_ exciton in 2D-WS_2_ under excitation with 4 μJ·cm^−2^ density are distinguished from those measured under higher and lower pump densities. Interestingly, this pump-density corresponds to the inflection point of the X_B_ exciton plot in Figure 3a. A possible explanation of this difference is that at this pump-density, the distribution of photogenerated electrons and holes of X_B_ exciton reaches an equilibrium between those decaying through trapping by defects and those decaying through direct electron-hole recombination.

An examination of the individual exponential components indicates that for both monolayers, the two excitons X_A_ and X_B_ decay through three different time scales. A fast process is described by $t_{1}$ (which has values ranging from a few hundred femtoseconds to a few picoseconds), a second process characterized by $t_{2}$ that is in the order of tens of picoseconds, and a much slower process described by $t_{3}$ with values reaching hundreds of picoseconds. This agrees with previous reports, which indicated that excitons in 2D-TMD monolayers decay through fast and slow pathways [20,21,22,23,24]. Previous studies of temperature and pump fluence-dependent dynamics indicated that exciton thermalization and relaxation happen within the first few picoseconds after excitation, but the defect-assisted exciton recombination takes place over longer time-scales [21,22]. Consequently, we assign the first component to thermalization and relaxation of excitons and free charge carriers. The second and third components, which vary from tens to hundreds of picoseconds, are attributed to the DAR process. Based on this assignment, we introduce an average time-constant for the DAR process, calculated as: $T_{DAR}=\left(A_{2}t_{2}+A_{3}t_{3}\right)/\left(A_{2}+A_{3}\right)$. Since the amplitudes $A_{i}$ are directly related to the excited population, the trends of $A_{i}$ with increasing pump density can provide information on the dominant decay pathway for excitons. In the case of exciton X_A_—in both materials, at the lowest pump fluences—about 70% of the excited population decays with the $t_{1}$ time-constant. As the excitation density increases, $A_{1}$ decreases, whereas $A_{2}$ and $A_{3}$ increase. This is surprising, because one expects that at high pump densities, the percentage of the population decaying through multiexciton effects (fast process) increases [6,26,36,38]. A possible interpretation of this behavior is that there are too many defect states available, and as the density of photogenerated excitons increases, the DAR pathway is favored.

**Figure 5:**
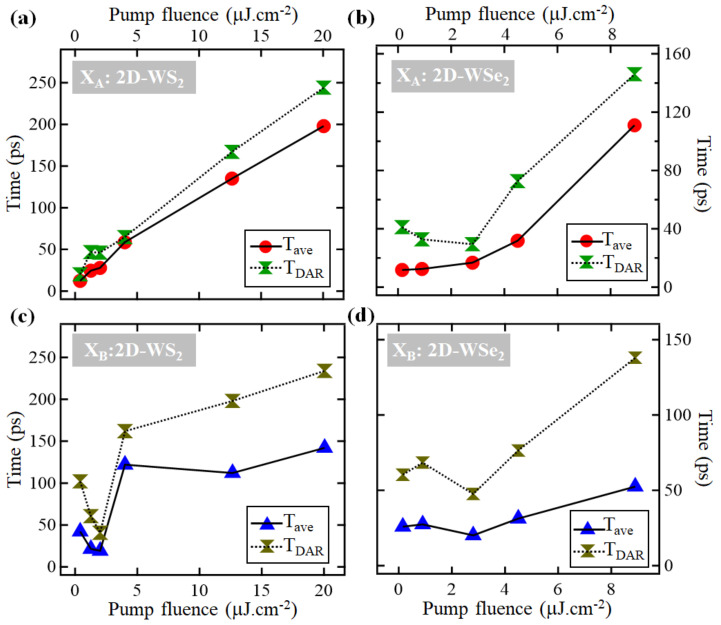
Dependence of $T_{ave}$ and $T_{DAR}$ of the depletion recovery dynamics of excitons on the pump fluence: (**a**) For exciton X_A_ in 2D-WS_2_; (**b**) For exciton X_A_ in 2D-WSe_2_; (**c**) For exciton X_B_ in 2D-WS_2_; (**d**) For exciton X_B_ in 2D-WSe_2_.

The dependence of $T_{ave}$ and $T_{DAR}$ of excitons X_A_ and X_B_ on the excitation density is shown in Figure 5. For the X_A_ exciton in 2D-WS_2_, $T_{ave}\left(X_{A}\right)$ and $T_{DAR}\left(X_{A}\right)$ increase almost linearly with increasing pump density, but in the case of the X_B_ exciton, the dependence is not linear. $T_{ave}\left(X_{B}\right)$ and $T_{DAR}\left(X_{B}\right)$ decrease as the pump fluence increases reaching ~2 μJ·cm^−2^, then they increase and reach a plateau when the fluence is above ~4.2 μJ·cm^−2^. In the case of 2D-WSe_2_, with the exception of the first two lowest pump densities, $T_{ave}$ and $T_{DAR}$ of both excitons X_A_ and X_B_ increase almost linearly with increasing pump density, and they do not reach a plateau at high pump densities.

The inflection points in the plots that describe the dependence of $T_{ave}$ and $T_{DAR}$ on the pump density, shown in Figure 5, coincide with the pump densities at which the exciton depletion signals reach their maxima (saturation) shown in Figure 3. In general, when a material reaches its saturated absorption, the trap states fill up first; consequently, $T_{DAR}$ should remain steady at some values. The fact that $T_{DAR}\left(X_{A}\right)$ in both monolayers and $T_{DAR}\left(X_{B}\right)$ in 2D-WSe_2_ continued increasing even after the pump densities reached the values of saturation (contrary to $T_{DAR}\left(X_{B}\right)$ in 2D-WS_2_, which reached a maximal value) suggests that in 2D-WS_2_, there are more trap-states near the X_A_ exciton, but in 2D-WSe_2_, they are located evenly near X_A_ and X_B_ excitons. An alternative explanation of the similar dependence of $T_{DAR}\left(X_{A}\right)$ and $T_{DAR}\left(X_{B}\right)$ on the pump-density in 2D-WSe_2_ may be attributed to the accuracy of the ground-state depletion signal in describing the dynamics of high energy excitons. For instance, in 2D-TMD monolayers in general, an excitation with photon-energies sufficient for generating exciton X_A_ but not sufficient for creating exciton X_B_ in pump-probe experiments, a depletion of the X_B_ exciton ground state is observed despite the fact that exciton X_B_ is not actually created [31,39]. This can be explained by the fact that when the X_A_ exciton is generated, the V_1_ level in the VB is partially depleted, which allows electrons at the V_2_ level (the ground-state level of exciton X_B_) to make transitions to the V_1_ level; and consequently, a depletion of the X_B_ exciton is observed despite the fact that this exciton is not created. Previous transient absorption studies have reported that the two SOS excitons in 2D-TMDs show correlated interexcitonic dynamics [34]. This effect manifests more importantly in cases when the excitation energy is sufficient for depleting levels deeper than V_2_ in the VB. Indeed, the excitation energy used in our study (~3.1 eV) depletes energy levels in the VB deeper than V_2_ in the case of 2D-WSe_2_ than in the case of 2D-WS_2_. Consequently, the depletion signal of exciton X_B_ observed in the case of 2D-WSe_2_ is less representative of the dynamics of exciton X_B_ than that in the case of 2D-WS_2_.

## 4. Conclusions

In summary, we investigated the dependence of exciton dynamics in 2D-WS_2_ and 2D-WSe_2_ monolayers on the pump-density using transient absorption spectroscopy. The exciton decay is measured through monitoring the exciton depletion recovery dynamics. These measurements indicated that the excited population of excitons and free charge carriers decays through a fast component during the first few picoseconds, and the remaining population decays through a slower pathway that takes place from ~tens to ~hundreds of picoseconds. The slow decay channel is attributed to the DAR process. Furthermore, the results indicated that the amplitude-weighted average lifetimes of either excitons X_A_ and X_B_ are driven by the DAR pathway. Additionally, in the 2D-WS_2_ monolayer, the defect states near the X_B_ exciton fill up before those near the X_A_ exciton; however, in the 2D-WSe_2_ monolayer, the defect states fill up similarly. This difference in exciton dynamics, despite the fact that the two monolayers are similar, may be due to one material having more defects (formed during the synthesis) than the other. These findings deepen our understanding of the process of exciton decay in 2D-TMD materials, and they may trigger additional investigations not only to understand the effect of the DAR process on the lifetime of excitons, but also the partition of the decay channel between X_A_ and X_B_ excitons and other higher energy excitons.

## Figures and Tables

**Table 1:** List of the fit parameters for the depletion recovery signal of excitons X_A_ and X_B_, upon excitation of the 2D-WS_2_ monolayer at ~3.1 eV with different densities. $A_{i}$ and $t_{i}$ are the amplitudes and time constants of the exponential component *i*. The amplitude-weighted average depletion recovery times $T_{ave}$ are calculated as $\sum_{i}A_{i}t_{i}$, and $T_{DAR}$ are calculated as $\left(A_{2}t_{2}+A_{3}t_{3}\right)/\left(A_{2}+A_{3}\right)$.

PP(μJ·cm^−2^)	0.4	1.3	2.0	4.0	13	20
*A*_1_(%): X_A_; X_B_	71; 62	48; 66	39; 53	10; 27	19; 43	18; 55
*t*_1_(ps): X_A_; X_B_	0.52; 0.90	0.43; 0.19	0.44; 0.33	1.75; 2.30	1.42; 0.78	1.30; 0.63
*A*_2_(%): X_A_; X_B_	15; 33	38; 24	46; 30	77; 67	60; 41	60; 34
*t*_2_(ps): X_A_; X_B_	14.6; 25.0	19.4; 14.4	19.4; 17.2	23.1; 31.5	47.6; 50.6	71.9; 116
*A*_3_(%): X_A_; X_B_	14; 5	14; 10	15; 17	13; 6	21; 16	22; 11
*t*_3_(ps): X_A_; X_B_	67.7; 610	121; 171	128; 81.8	312; 1624	508; 575	714; 598
***T_DAR_*(ps): X_A_; X_B_**	**40.2; 102**	**46.7; 60.5**	**46.1; 40.6**	**64.8; 162**	**167; 198**	**244; 234**
***T_ave_*(ps): X_A_; X_B_**	**12.2; 42.1**	**24.5; 21.4**	**27.8; 19.1**	**58.6; 122**	**135; 112**	**198; 142**

**Table 2:** List of the fit parameters for the depletion recovery signal of excitons X_A_ and X_B_, upon excitation of the 2D-WSe_2_ monolayer at ~3.1 eV with different densities. $A_{i}$ and $t_{i}$ are the amplitudes and time constants of the exponential component *i*. The amplitude-weighted average depletion recovery times $T_{ave}$ are calculated as $\sum_{i}A_{i}t_{i}$, and $T_{DAR}$ are calculated as $\left(A_{2}t_{2}+A_{3}t_{3}\right)/\left(A_{2}+A_{3}\right)$.

PP(μJ·cm^−2^)	0.15	0.90	2.80	4.50	8.90
*A*_1_(%): X_A_; X_B_	72; 57	64; 63	46; 59	59; 61	24; 64
*t*_1_(ps): X_A_; X_B_	0.34; 0.17	0.35; 0.30	1.70; 1.60	3.43; 1.61	2.81; 0.98
*A*_2_(%): X_A_; X_B_	15; 25	25; 29	48; 37	35; 36	61; 29
*t*_2_(ps): X_A_; X_B_	4.7; 1.72	9.20; 8.10	11.2; 13.3	21.0; 15.3	30.5; 30.4
*A*_3_(%): X_A_; X_B_	13; 18	11; 8	6; 4	6; 3	15; 7
*t*_3_(ps): X_A_; X_B_	83.3; 142	86.4; 287	175; 365	374; 811	614; 586
***T_DAR_*(ps): X_A_; X_B_**	**41.2; 60.4**	**32.8; 68.4**	**29.4; 47.6**	**72.7; 76.5**	**146; 138**
***T_ave_*(ps): X_A_; X_B_**	**11.8; 25.9**	**12.5; 27.4**	**16.8; 20.1**	**31.8; 31.3**	**111; 52.5**

## Data Availability

Data is contained within the article or supplementary materials. The data presented in this study are available in [Figure 1, Figure 2, Figure 3, Figure 4 and Figure 5 and Table 1 and Table 2 in the main text, and Figure S1 and Tables S1 and S2 in the supplementary materials].

## Supplementary Materials

The following are available online at https://www.mdpi.com/2079-4991/11/3/770/s1, Figure S1: Multi-peak fitting of a transient absorption spectrum collected 10 ps following exciton of the monolayer of 2D-WS_2_ (a), and 2D-WSe_2_ (b). The individual components of the fits are shown in the bottom panels; Table S1: List of the muti-Gaussian peak fit of a transient absorption spectrum measured at 10 ps following excitation of the 2D-WS_2_ monolayer at 3.1 eV with 4 μJ·cm^−2^. I (mOD): peak amplitude; C_0_ (nm): peak center; w(nm): full width at half maximum of the peak; Table S2: List of the muti-Gaussian peak fit of a transient absorption spectrum measured at 10 ps following excitation of the 2D-WSe_2_ monolayer at 3.1 eV with 2.8 μJ·cm^−2^. I (mOD): peak amplitude; C_0_ (nm): peak center; w(nm): full width at half maximum of the peak.
