# Supplementary material for: Filling Exciton Trap-States in Two-Dimensional Tungsten Disulfide (WS2) and Diselenide (WSe2) Monolayers
Source: Nanomaterials (Basel). 2021 Mar 18;11(3):770. doi: 10.3390/nano11030770 (PMC8002918; doi:10.3390/nano11030770)
Supplement: Supplementary file 1 [file nanomaterials-11-00770-s001.pdf]

nanomaterials

MDPI

Supplementary Materials

Filling Exciton Trap-States in Two-Dimensional Tungsten Disulfide (WS_2) and Diselenide (WSe_2) Monolayers

Zeynep Ezgi Eroglu [1], Dillon Contreras [1], Pouya Bahrami [1], Nurul Azam [2], Masoud Mahjouri-Samani [2] and Abdelaziz Boulesbaa [1,*]

[1] Department of Chemistry & Biochemistry, California State University, Northridge, 18111 Nordhoff Street, Northridge, CA 91330, USA; zeynep.eroglu.568@my.csun.edu (Z.E.E.); dillon.contreras.3@my.csun.edu (D.C.); pouya.bahrami.60@my.csun.edu (P.B.)

[2] Department of Electrical and Computer Engineering, Auburn University, Auburn, AL 36849, USA; mna0018@auburn.edu (N.A.); mzm0185@auburn.edu (M.M.-S.)

* Correspondence: aboules@csun.edu

Spectral analysis of transient spectra:

To spectrally resolve the different components of the positive induced absorption (IA) band observed in Figure 2 in the Main Text, we conducted a multi-Gaussian peak fit of transient absorption spectra taken at 10 ps time-delays. This time-delay is chosen because by 10 ps, the IAs resulting from exciton line broadening due to many-body interactions have recovered [1–4]. The returned fitting parameters are listed in Table S1 for 2D-WS_2 and Table S2 for 2D-WSe_2.

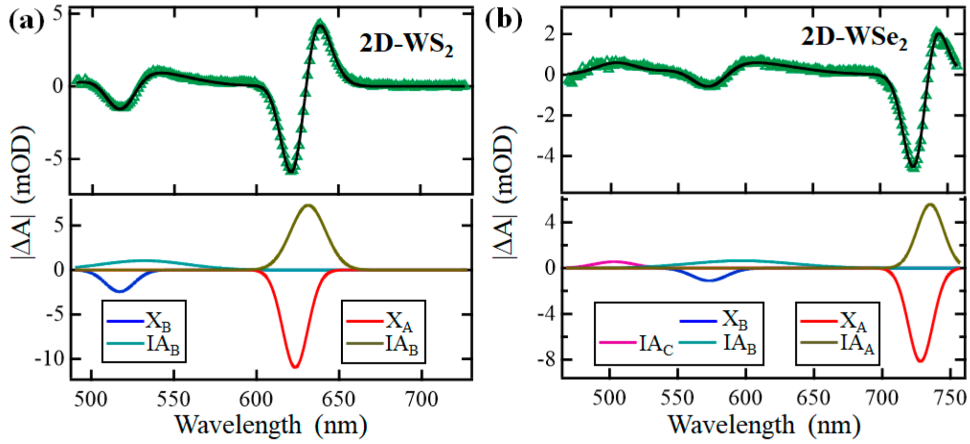

Figure S1. Multi-peak fitting of a transient absorption spectrum collected 10 ps following exciton of the monolayer of 2D-WS_2 **(a)**, and 2D-WSe_2 **(b)**. The individual components of the fits are shown in the bottom panels.

In the case of 2D-WS_2, we identified four compenents; two negative peaks corresponding to depletions of excitons X_A and X_B, and two positive features IA_A near X_A and IA_B near X_B. In the case of 2D-WSe_2, and additional IA_C was indentified. We note that the probe used in both sample starts at 480 nm, which allows us to capture the IA_C, in 2D-WS_2, one expects the existance of a similar IA at shorter wavelengths that we can not capture using our probe.

Table S1. List of the muti-Gaussian peak fit of a transient absorption spectrum measured at 10 ps following excitation of the 2D-WS_2 monolayer at 3.1 eV with 4 J·cm^{-2}. I (mOD): peak amplitude; C_0 (nm): peak center; w(nm): full width at half maximum of the peak.

	X_B	IA_B	X_A	IA_A
I (mOD)	−2.45	+1.05	−11.2	+7.4
C_0 (nm)	517.2	531.9	623.4	631.0
w (nm)	20.5	61.4	18.1	24.8

Table S2. List of the muti-Gaussian peak fit of a transient absorption spectrum measured at 10 ps following excitation of the 2D-WSe_2 monolayer at 3.1 eV with 2.8 J·cm^{-2}. I (mOD): peak amplitude; C_0 (nm): peak center; w(nm): full width at half maximum of the peak.

	IA_C	X_B	IA_B	X_A	IA_A
I (mOD)	+0.56	−1.10	+0.65	−8.15	+5.55
C_0 (nm)	503.4	573.1	596.5	728.1	735.2
w (nm)	35.1	29.7	89.3	21.1	22.9

Supplementary References

1. Sim, S.; Park, J.; Song, J.-G.; In, C.; Lee, Y.-S.; Kim, H.; Choi, H. Exciton dynamics in atomically thin MoS_2: Interexcitonic interaction and broadening kinetics. *Phys. Rev. B* **2013**, 88, 075434.
2. Yuan, L.; Huang, L. Exciton dynamics and annihilation in WS2 2D semiconductors. *Nanoscale* **2015**, *7*, 7402–7408.
3. Sun, D.; Rao, Y.; Reider, G. A.; Chen, G.; You, Y.; Brezin, L.; Harutyunyan, A. R.; Heinz, T. F. Observation of Rapid Exciton−Exciton Annihilation in Monolayer Molybdenum Disulfide**.** *Nano Lett.* **2014**, *14***,** 5625–5629.
4. Cunningham, P. D.; McCreary, K. M.; Jonker, B. T. Auger Recombination in Chemical Vapor Deposition-Grown Monolayer WS_2. *J. Phys. Chem. Lett.* **2016**, 7, 5242–5246.
